# Green haemodialysis: comparison of dialysis bags versus fresenius granumix at the AOU Policlinico di Modena, Italy

**DOI:** 10.1007/s40620-025-02416-0

**Published:** 2025-11-01

**Authors:** James Larkin, Gaetano Alfano, Giulia Ligabue, Rodrigo Martínez Cadenas, Karin G. F. Gerritsen, Abass Fehintola, Gabriele Donati, Brett Duane

**Affiliations:** 1 School of Dental, Child and Public Health, Trinity College Dublin, Dublin, Ireland; 2 Department CHIMOMO, University of Modena and Reggio Emilia, Modena, Italy; 3 Universidad Autonoma de Madrid, Madrid, Spain; 4 Nephrology, Dialysis and Kidney Transplant Unit, University Hospital Policlinico of Modena, Modena, Italy; 5 University Medical Centre Utrecht, Utrecht, Netherlands

**Keywords:** Haemodialysis, End stage kidney disease, Environment, Sustainability, Life cycle assessment, Acid concentrate bags, Fresenius granumix plus system, Dialysate, Resource consumption, Waste generation, Carbon footprint, Water use

## Abstract

**Background:**

Haemodialysis (HD) has a significant environmental footprint due to its high resource consumption  and waste generation. A key component is dialysate production, which typically involves mixing water, bicarbonate, and a single-use acid concentrate bag for each session. An alternative method is the central delivery of acid concentrate, prepared on-site using dry powder formulations. This study compares the environmental impact of these two delivery systems, focusing on waste reduction, elimination of single-use plastic, and decreased transport-related emissions.

**Methods:**

A detailed life cycle assessment (LCA) was carried out using OpenLCA and the Ecoinvent v3.1 database to compare the traditional acid concentrate bag system with the central delivery system (Granumix, Fresenius, Germany). Data on material inputs, energy use, waste generation, and transportation were collected. Flow diagrams captured the full life cycle of each method, and various environmental indicators were analysed, including climate change, acidification, and ecotoxicity.

**Results:**

The Granumix system demonstrated considerably lower environmental impact than traditional bag use. It reduced climate change potential by 30% (CO₂-equivalent) and freshwater ecotoxicity by 15%. Additional benefits were observed in acidification, resource use, and overall emissions, indicating the system’s broader environmental advantages.

**Conclusions:**

The central delivery of acid concentrate using the Granumix system is a more sustainable option for HD, significantly reducing environmental burdens across several categories. These findings underscore the value of adopting innovative delivery models to make essential healthcare treatments more environmentally sustainable.

**Graphical abstract:**

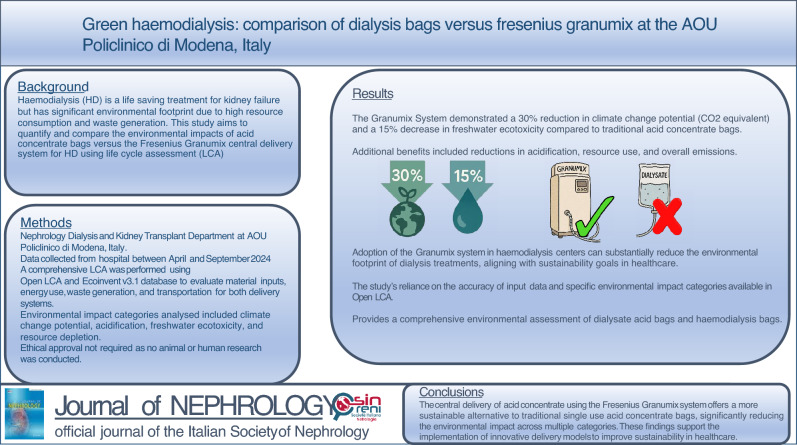

**Supplementary Information:**

The online version contains supplementary material available at 10.1007/s40620-025-02416-0.

## Introduction

Haemodialysis (HD) is a lifesaving treatment for patients with kidney failure [1]. In 2017, estimates indicated that 3.9 million people with kidney failure were being treated with Kidney Replacement Therapy (KRT) globally[1].This prevalence is projected to increase year over year due to the ageing population, an increase in lifestyle diseases such as hypertension and diabetes, and greater access globally to health care treatment [2]. HD remains the commonest form of KRT, accounting for approximately 89% of all dialysis treatments [3].

The environmental impact of HD is significant; it is considered one of the most energy-intensive procedures in healthcare, particularly due to its high resource consumption and waste generation [2]. The substantial energy demands of HD arise from multiple aspects of the treatment process, including the production of dialysate, which involves mixing pure or ultrapure, deionised water with both an acid and a base concentrate. The HD machine prepares dialysis fluid by mixing manufacturer-supplied concentrates with treated water in precise proportions to achieve the desired composition.

Currently, there are several methods for delivering acid concentrate to dialysis machines:

i. concentrated liquid in individual plastic containers (bags or canisters),
ii. individual cartridges of powder,
iii. semi-dry concentrates delivered in reusable barrels for bulk automated reconstitution [4],
iv. premixed acid pumped into large on-site storage tanks, and
v. bags of dry powder for bulk manual reconstitution.

Options (i) and (ii) allow for individualisation of prescriptions, making them suitable for patient-specific treatment adjustments. In contrast, options (iii), (iv), and (v) are used in central delivery systems and do not allow for easy prescription individualisation.

In this study, we focused only on the acid delivery systems available at the AOU Policlinico di Modena—specifically option (i), represented by single-use acid bags (Baxter Softpak 3.5 L), and option (iii), represented by the Granumix system. Other international configurations were not assessed due to lack of availability at the study site.

The primary advantage of (i) and (ii) is the ability to customise the dialysate to suit a patient's specific needs; but this advantage in clinical care is offset by its significant contribution to the environmental impact in terms of plastic waste and discharge of unused acid concentrate [3, 5].

In contrast, the use of centralised systems such as the B. Braun EcoMix or Fresenius Medical Care Granumix Plus represents a significant step towards reducing the environmental impact of haemodialysis. These systems automate the production and distribution of acid concentrate directly to HD machines within a facility, eliminating the need for individual single-use containers [6]. The Granumix system uses reusable Diamix powder containers, each of which can be reused up to ten times before being recycled, thereby significantly reducing plastic waste. Additionally, the system relies on pre-treatment filters and other minor consumables that are replaced periodically and have been included in our life cycle analysis. The filter (FX Cordiax 100) contributes minimally to material use and waste due to its long service life and infrequent replacement (3 used simultaneously, changed once a year). By reducing plastic use, minimising chemical waste, and lowering transport-related emissions, the Granumix system offers a more sustainable approach to dialysate preparation and delivery.

Life Cycle Assessment (LCA) is a method used to evaluate the environmental impacts associated with all stages of a product or process, from raw material extraction through manufacturing, usage, and disposal or recycling. Life cycle assessment generates results across various environmental categories, such as carbon footprint, water use, energy consumption, acidification, eutrophication, and toxicity. These results are typically complex and expressed in units specific to each impact category [7]. To facilitate interpretation, results are often normalised, converting them into dimensionless scores that allow straightforward comparisons across categories. Normalisation involves scaling results relative to average environmental impacts caused by a standard reference, often referred to as the "average person", enabling readers to easily understand whether a particular impact is higher or lower than average [8].

## Aim of the study

This study aims to evaluate the environmental impact of acid concentrate delivery systems in haemodialysis by conducting a comprehensive life cycle assessment comparing traditional single use acid concentrate bags with the Fresenius Granumix system. By analysing key environmental impact categories, including climate change potential, acidification, freshwater ecotoxicity, and resource depletion, this research seeks to quantify the sustainability benefits of central acid concentrate preparation and distribution. The study further explores potential areas for improvement in dialysis sustainability, highlighting strategies to reduce emissions, waste generation, and resource consumption. The findings aim to provide healthcare providers, policymakers, and manufacturers with data-driven insights to promote greener dialysis practices and support the transition towards more sustainable kidney care solutions.

## Methods

This study uses a comprehensive life cycle assessment to evaluate the environmental sustainability of two acid concentrate delivery methods used in haemodialysis: traditional single-use acid concentrate bags and the Fresenius Granumix central delivery system. Life cycle assessment is a widely adopted method for quantifying the environmental impacts associated with a product or process across its full life cycle—from raw material extraction to end-of-life disposal. Data collection and modelling were carried out between April and September 2024 at the AOU Policlinico di Modena in Italy. The study involved detailed process mapping, material inventory, energy and water usage, and waste management assessment for both systems.

### Creating flow diagrams

The first step involved developing a detailed flow diagram to visualise the entire life cycle of both dialysis methods. The flow diagram serves as a graphical representation of the processes and stages involved in the production, use and waste of the two dialysis methods. All processes involved in the life cycle of methods were identified, including materials, products and waste.

**Fig. 1:**
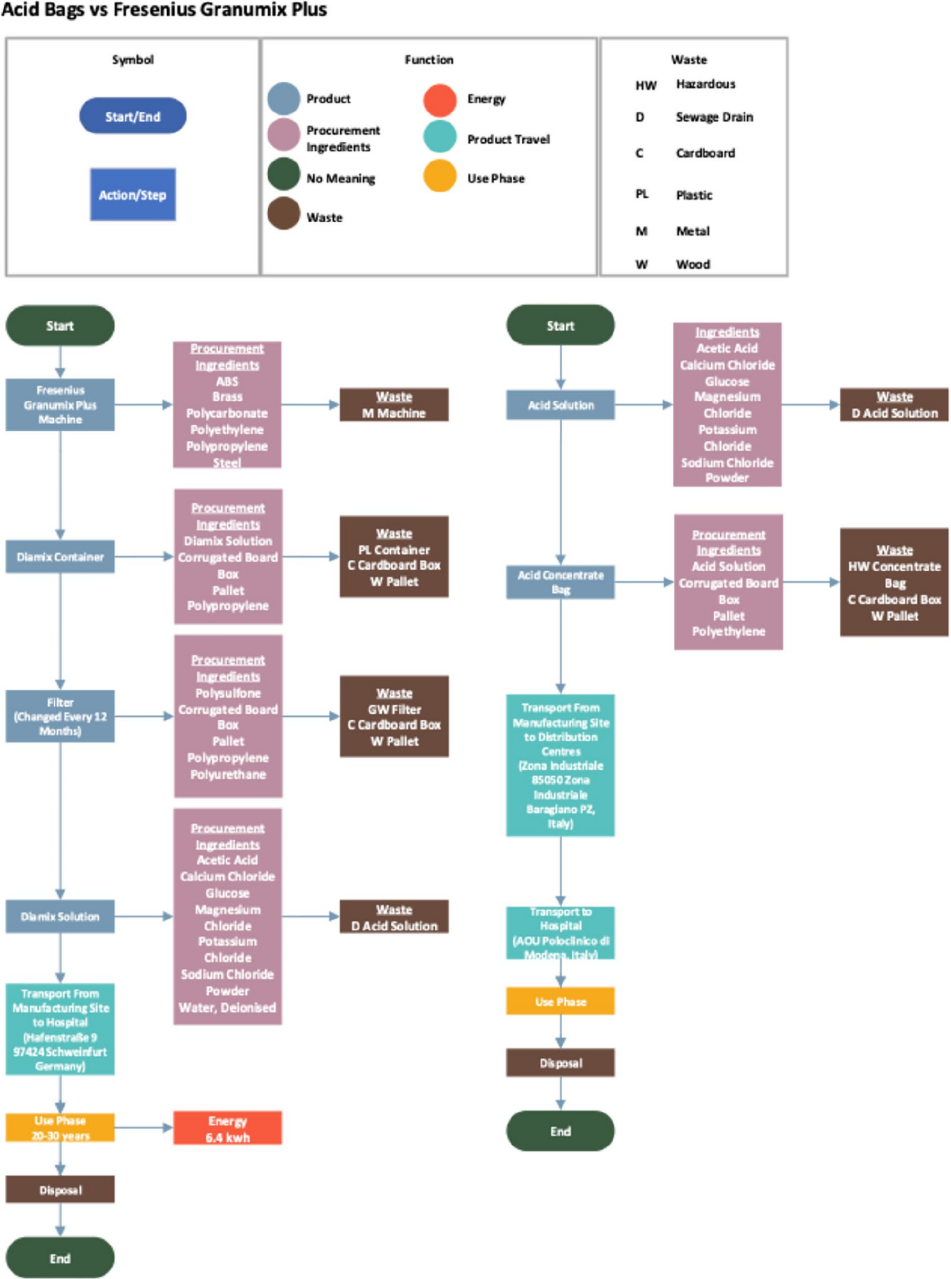
Flow Diagram of acid bags vs Acid Concentrate

Each process was mapped out. Flow diagrams were created using a professional page layout software, Visio [9] – See Fig. 1

The flow diagrams were verified through discussion and consultation with clinical staff and dialysis experts to ensure accuracy.

### Data collection

Data were collected from the Nephrology Dialysis and Kidney Transplant Department at the AOU Policlinico di Modena, Italy, and from a previous study on Acid concentrate by nephrologists and researchers at the same hospital [8].

Collected data included the number of acid concentrate bags used annually, the volume of dialysate produced by the Granumix machine, the amount of waste generated, including packaging waste, acid solution and other related items and materials. Resource consumption data, including energy and water usage were also collated.

Inputting data into OpenLCA.

The material weights and types alongside the process flow diagrams were input into OpenLCA [10]. This involved compiling all relevant data for both dialysis methods.

The data were used to model the life cycle of both dialysis methods.

Impact assessment was conducted using various environmental impacts such as climate change, ecotoxicity, resource use, water use in OpenLCA alongside the Eco Invent database [11]. This step involved calculating the environmental impacts across various impact categories, such as global warming potential, ecotoxicity and acidification.

Bicarbonate cartridges were excluded from this life cycle assessment as their use and environmental impact were identical across both systems and therefore would not affect the comparative results.

The full data set for this study can be found at Larkin, J. (2025). Green Haemodialysis: Comparison of Dialysis Bags Versus Fresenius Granumix Water Recycling System at the AOU Policlinico di Modena, Italy (Version v1) [Data set]. Zenodo. 10.5281/zenodo.14610055.

### Functional unit

The functional unit for this study is defined as the amount of dialysate required for one haemodialysis treatment for a single patient. Each session was assumed to last 4 h with a dialysate flow rate of 500 mL/min**,** resulting in a total of 120 L of dialysate per treatment. This standardised prescription allows for comparability across haemodialysis units and reflects common clinical practice.

### Impact categories in life cycle assessment

This study used OpenLCA to analyse a range of environmental impact categories relevant to dialysis delivery systems. These included:

- Acidification potential, measured in mol H⁺-eq, which reflects the emission of substances (e.g., SO₂, NOₓ, NH₃) that increase the acidity of soil and water bodies, potentially harming ecosystems.
- Eutrophication, measured in kg P-eq or kg N-eq, refers to the nutrient enrichment of water bodies (freshwater, marine, or terrestrial) that can lead to excessive algal growth and oxygen depletion.
- Human toxicity (carcinogenic and non-carcinogenic), measured in comparative toxic units for humans, estimates the risk to human health from exposure to harmful substances over the product lifecycle.
- Freshwater ecotoxicity, measured in comparative toxic units for ecosystems, evaluates the potential of emissions to cause toxic effects in aquatic organisms.
- Other categories include climate change (kg CO₂-eq), ozone depletion (kg CFC-11-eq), particulate matter formation (disease incidence), water use (m^3^ world eq. deprived), land use, and resource depletion (e.g., kg Sb-eq for metal use).

A full glossary of all impact categories, including definitions and units, is provided in the supplementary material (Supplementary Material S1: life cycle assessment Impact Categories).

### System boundaries

**Fig. 2:**
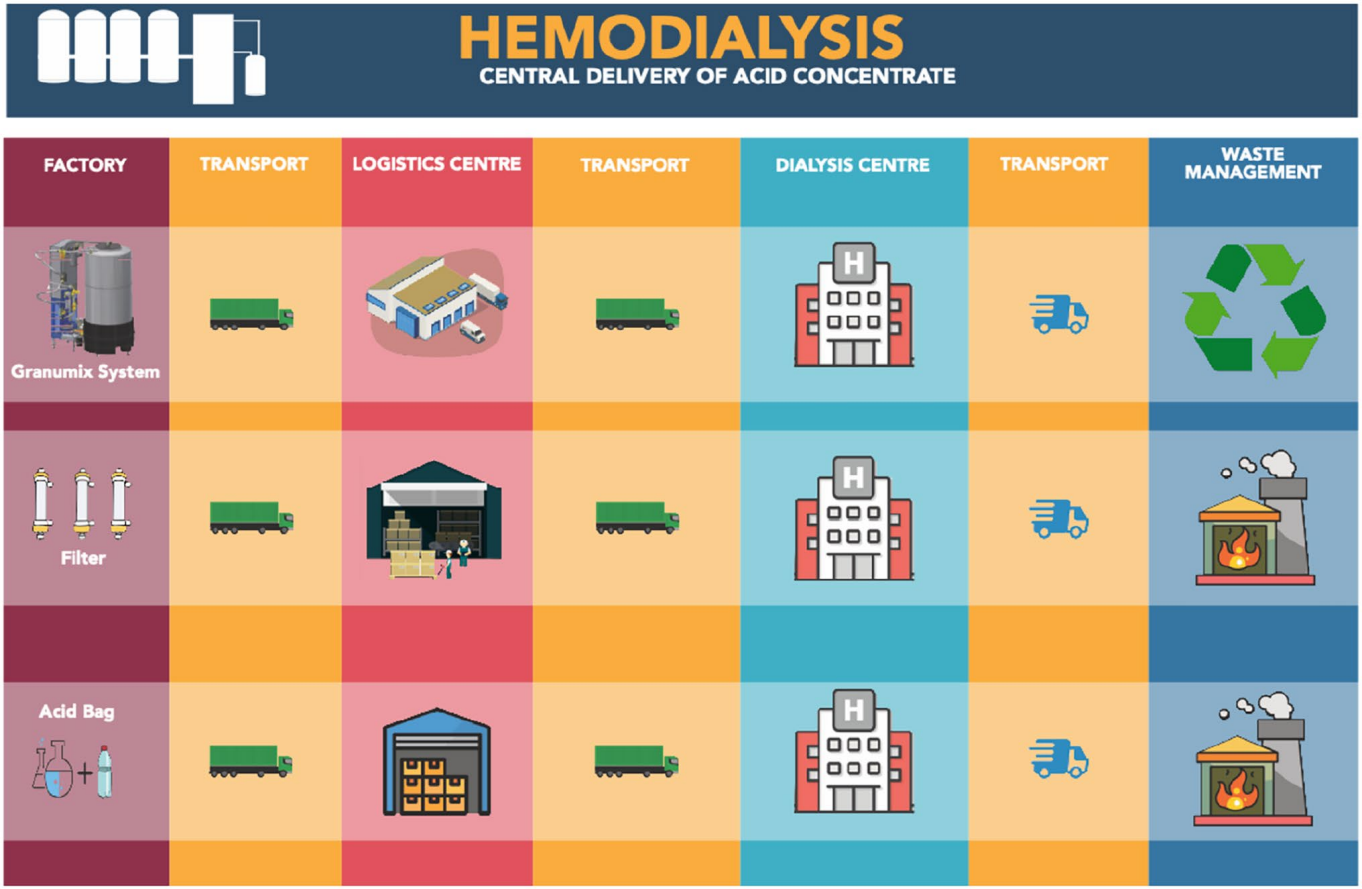
System boundaries for Acid Concentrate Bags and the Fresenius Granumix System. The system boundary shows the “cradle to grave” analysis of the life cycle assessment

Figure 2 outlines the system boundaries, covering the full life cycle of traditional acid concentrate bags and the Fresenius Granumix system, from raw material extraction to end-of-life waste. For acid bags, materials include plastics, acid solution chemicals, and packaging (3.8–4.5 L per bag). Granumix materials include metals, plastics, and other system components. The filter, including its material composition, packaging, transport distances, and replacement frequency, was included in the life cycle inventory for the Granumix system to ensure a complete and representative environmental assessment.

Manufacturing includes plastic bag and acid solution production for the bags, and full system and component assembly for the Granumix. Transportation covers raw material delivery, product distribution, and maintenance-related transport for both systems. Waste transport is also included.

Both the use and waste phases are assessed, including disposal of used bags and solution, as well as waste from Granumix components.

### Product manufacture

The product materials and weight information were retrieved from the previous study which investigated the difference between acid concentrate bags and the Granumix system with regard to the quantity of each material and the amount of waste generated [9]. The manufacturing processes that were used in the production of all the products were found through product safety data sheets and assumptions. These have all been referenced at Larkin, J. (2025). Green Haemodialysis: Comparison of Dialysis Bags Versus Fresenius Granumix Water Recycling System at the AOU Policlinico di Modena, Italy (Version v1) [Data set]. Zenodo. 10.5281/zenodo.14610055. The energy usage of the machinery that was used to manufacture the products was provided by Eco Invent.

### Transportation

Transportation distances were calculated from the manufacturing site to the AOU Policlinico di Modena using Google Maps for road transport and Fluentcargo.com for shipping distances. These tools provided the most geographically direct and commonly used routes for freight transport, based on available infrastructure. Actual routes may vary depending on specific logistics provider operations, but the shortest and fastest available routes were used as a conservative estimate for environmental modelling.

Information on modes of transport (e.g., truck, ship) was obtained from product manufacturers where possible. In instances where exact transport logistics were unavailable, reasonable assumptions were made based on standard industry practices for medical device distribution, considering typical transport modes, packaging formats, and the origin–destination pairing.

### Waste

To evaluate the environmental impact of waste, data were collected from hospital administrative staff on the hospital’s waste management processes. Healthcare waste and recycled materials were the main focus. Hospital admin staff were able to identify waste management providers, and the waste processes for the acid concentrate bags as well as the recycling of the Granumix container. Waste was categorised into healthcare waste and recyclable materials to assess their environmental impacts. The quantity of unused acid solution was estimated based on observational data provided by the nursing staff at AOU Policlinico di Modena, with an average of 0.4 L discarded per bag.

In Italy, acid concentrate bags are classified as hazardous medical waste due to potential chemical contamination and are typically incinerated as part of national healthcare waste protocols. This may differ from practices in other countries, where such waste might be considered non-hazardous and treated via alternative methods such as autoclaving or landfill disposal. This has implications for the generalisability of toxicity and waste-related results in the life cycle assessment.

### Data analytics

The process involved entering the collected data into the OpenLCA software that incorporated all aspects of the products with the cradle to grave approach [12]. A detailed life cycle model was created using various impact categories such as acidification, climate change, and ecotoxicity to calculate environmental impacts across multiple categories, including areas such as global warming potential, resource depletion, and human health impacts. The analysis identified and quantified the most significant environmental contributors between acid concentrate bags and the Fresenius Granumix system. Detailed assessments of each process and material involved were included.

## Results

Life cycle assessment results across 15 impact categories

The life cycle assessment compared the environmental impacts of acid concentrate bags and the Fresenius Granumix central delivery system across 15 impact categories. The results are presented in Table 1. Across all categories, the Granumix system demonstrated reduced environmental impacts compared to traditional single-use acid concentrate bags.

**Table 1:** LCA Results Across 15 Impacts

Impact Categories	Unit	Acid Bag	Granumix system	% Reduction
Acidification	mol H + -Eq	2.36E-02	1.97E-02	17%
Climate change	kg CO2-Eq	3.74E + 00	2.63E + 00	30%
Ecotoxicity: freshwater	CTUe	5.70E + 01	4.90E + 01	14%
Eutrophication: freshwater	kg P-Eq	6.29E-03	6.12E-03	3%
Eutrophication: marine	kg N-Eq	8.29E-03	7.33E-03	12%
Eutrophication: terrestrial	mol N-Eq	5.68E-02	4.78E-02	16%
Human toxicity: carcinogenic	CTUh	2.35E-09	1.60E-09	32%
Human toxicity: non-carcinogenic	CTUh	5.42E-08	4.60E-08	15%
Ionising radiation: human health	kBq U235-Eq	2.75E-01	2.42E-01	12%
Land use—soil quality index	dimensionless	6.99E + 01	2.73E + 01	61%
Material resources: metals/minerals	kg Sb-Eq	7.87E-05	7.68E-05	3%
Ozone depletion	kg CFC-11-Eq	4.60E-07	4.04E-07	12%
Particulate matter formation	disease incidence	2.20E-07	1.56E-07	29%
Photochemical ozone formation	kg NMVOC-Eq	1.29E-02	9.55E-03	26%
Water use	m3 world eq. deprived	4.12E + 00	3.97E + 00	4%

The climate change impact, measured in kg CO2-equivalent, showed that the traditional acid concentrate bags contribute significantly more to global warming (3.74 kg CO2-eq) compared to the Granumix system (2.63 kg CO2-eq). A 29.83% reduction in greenhouse gas emissions was seen when using the Granumix system, primarily due to the elimination of the need for the production, transportation, and waste of large quantities of single-use acid concentrate bags.

Extrapolating these findings to the AOU Policlinico di Modena dialysis unit, which treats, on average, 251 patients three times per week, the total estimated CO₂-equivalent reduction from implementing the Granumix system is approximately 43.5 tonnes per year. This cumulative impact highlights the substantial environmental benefit achievable at the facility level, reinforcing the value of transitioning to central acid delivery systems to reduce the carbon footprint of haemodialysis at scale.

In terms of acidification potential, the results reveal that the Granumix system also has a lower impact (0.0197 mol H⁺-Eq) compared to acid concentrate bags (0.0236 mol H⁺-Eq), with the reduction attributed to the minimised use of packaging materials and fewer emissions associated with transportation.

Concerning ecotoxicity, particularly in freshwater ecosystems, the Granumix system demonstrates a lower impact (49.03 comparative toxic units for ecosystems) compared to acid concentrate bags (57.03 comparative toxic units for ecosystems), i.e., a reduction of 14.02%. The reduced release of harmful substances during the production and waste stages of the Granumix system (compared to the production of single-use concentrate bags) likely contributes to this improvement., Both freshwater (0.00612 vs 0.00629 kg P-Eq) and marine (0.00733 vs 0.00829 kg N-Eq) eutrophication impacts are slightly lower for the Granumix system. The reduced nutrient release into aquatic systems highlights the environmental benefits of the Granumix system.

In terms of human toxicity, both carcinogenic (1.60E-9 vs 2.35E-9 comparative toxic units for humans) and non-carcinogenic (4.60E-8 vs 5.42E-8 comparative toxic units for humans) effects were lower for the Granumix system. These toxicity impacts arise primarily from the production of materials (e.g., plastics and chemicals) and end-of-life processes such as incineration, rather than the direct use of the systems during dialysis. The difference in toxicity scores reflects a lower release of harmful substances over the lifecycle of the Granumix system compared to single-use acid concentrate bags.

The analysis of resource use (7.68E-5 vs 7.87E-5 kg Sb-Eq) and ozone depletion potential (4.04E-7 vs 4.60E-7 kg CFC-11-Eq) also favours the Granumix system. Similarly, ozone depletion potential is lower for the Granumix system.

Water use, a critical environmental factor in dialysis, shows a notable reduction with the Granumix system, consuming 3.97 m^3^ world eq. deprived compared to 4.12 m^3^ for acid bags.

### Normalised analysis

* Y-axis represents dimensionless normalised environmental impact scores, scaled relative to the average global person’s annual contribution to each category (based on Ecoinvent v3.1 normalisation factors).

**Fig. 3:**
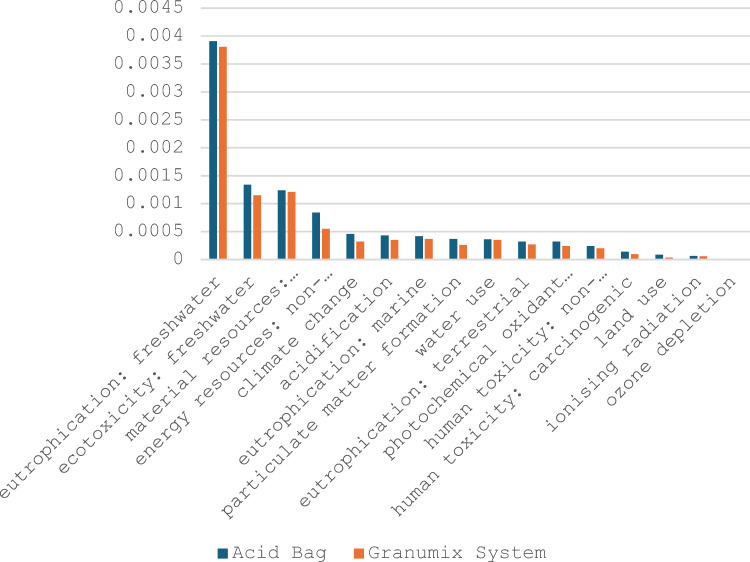
Normalised Scores Across All Impacts

Figure 3 compares the normalised environmental impacts of the Acid Bag and Granumix systems across multiple categories. **Freshwater eutrophication** and **ecotoxicity** were the highest impacts for both, with the Acid Bag showing consistently higher scores. This supports the environmental benefits of centralised systems like Granumix in dialysis care.

### Contribution analysis

**Fig. 4:**
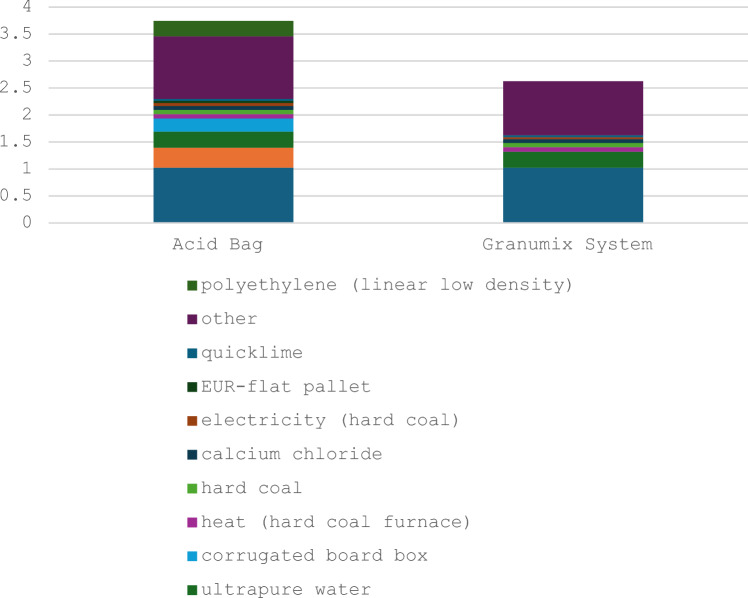
Contribution Analysis, Green House Gas (GHG) emissions

**Fig. 5:**
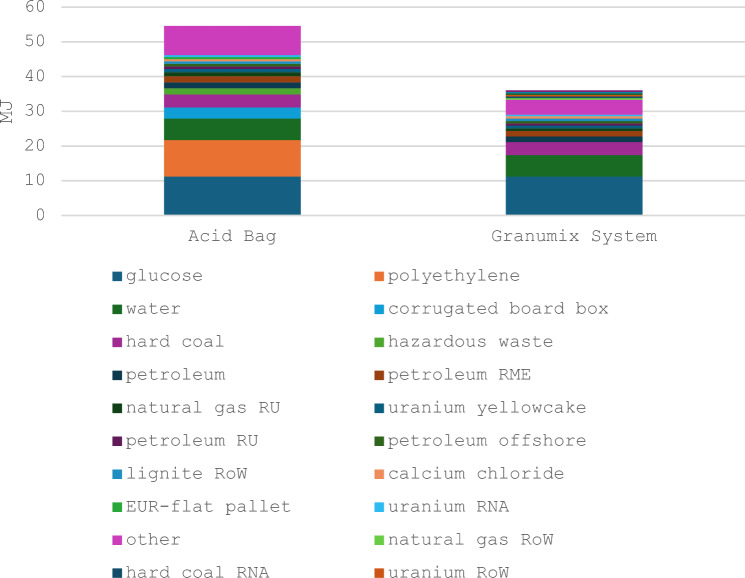
Contribution Analysis, Energy Resources

The life cycle assessment found that material production, transport, and waste management were the main impact drivers. The Acid Bag system had higher emissions due to single-use components, including polyethylene (0.285 kg CO₂-eq), packaging (0.240 kg CO₂-eq), and waste incineration (0.372 kg CO₂-eq). In contrast, Granumix uses reusable containers and local preparation, avoiding most single-use waste and reducing emissions across impact categories (Figs. 4–5, Appendix 3).

At AOU Policlinico di Modena, each Acid Bag generated 372 g CO₂-eq from waste incineration, plus 53.5 g from cleaning residues and 72.8 g from packaging—emissions that are avoided in Granumix. Recyclable Granumix containers produced just 0.020 kg CO₂-eq.

Energy use was dominated by glucose and ultrapure water production (11.21 MJ and 6.19 MJ). The Acid Bag system had added impacts from polyethylene (10.51 MJ), packaging (3.17 MJ), and hazardous waste (1.76 MJ)—again, avoided with Granumix. Small reductions were also seen in petroleum, gas, and uranium use, showing overall lower energy resource consumption for the Granumix system.

Contribution analysis figures for all environmental impact categories are provided in Appendix 3.

## Discussion

This study demonstrates that the centralised acid delivery system using the Fresenius Granumix provides a more sustainable alternative to single-use acid concentrate bags for haemodialysis. Through life cycle assessment, we found that the Granumix system reduces environmental impacts across multiple categories, including climate change potential, resource consumption, waste generation, and ecotoxicity.

Our findings align with a growing body of evidence supporting the environmental advantages of central acid delivery systems in dialysis. The Bradford NHS study reported substantial carbon footprint reductions following a similar transition to central acid delivery [13]. Beyond greenhouse gas emissions, our study expands the analysis by considering additional environmental categories such as acidification, freshwater ecotoxicity, and human toxicity. These broader findings highlight the environmental trade-offs often overlooked when focusing solely on carbon emissions.

The results indicate that dialysis centres seeking to reduce their environmental footprint can benefit from implementing centralised acid delivery systems. Key advantages include reductions in single-use plastic waste, packaging, and transport-related emissions. Furthermore, decreased hazardous waste generation can ease the burden on hospital waste management systems. As healthcare providers work toward decarbonisation targets, such procurement decisions can make a meaningful contribution to greener dialysis care.

Despite its environmental advantages, the Granumix system's production process still contributes to resource use and emissions, primarily due to the use of metals and plastics. Opportunities for further environmental improvement include incorporating recycled materials into system components [14], utilising bioplastics [15], and integrating renewable energy sources into manufacturing processes [16]. In addition, adopting circular economy principles, such as closed-loop recycling for Granumix components, could further minimise environmental burdens [17, 18].

While this study focused on the comparison between single-use acid concentrate bags and a dry powder-based central acid delivery system (Granumix), it is important to acknowledge that liquid centralised acid delivery systems represent another alternative used in dialysis facilities. These systems store premixed acid solution in large on-site tanks, eliminating single-use containers and reducing packaging waste, similar to the Granumix approach [19]. However, compared to dry powder-based systems, liquid central delivery may involve higher transport-related emissions due to the weight of transporting large volumes of liquid. Existing studies suggest that while liquid central delivery reduces waste generation, the overall environmental advantage compared to dry powder systems depends on factors such as transport distances, facility size, and local infrastructure [4, 6]. Further research directly comparing dry powder and liquid central delivery would be valuable to inform optimal sustainable procurement choices for haemodialysis centres.

It is important to acknowledge the limitations of this study. Life cycle assessment relies on the quality and accuracy of available input data, including material weights, transport distances, and process energy use. While reasonable assumptions were made where necessary, actual transport routes and energy mixes may vary across regions and facilities. The study is also limited to a single healthcare centre, which may affect the generalisability of results. Future research should explore life cycle assessments of alternative acid delivery methods, including emerging technologies or configurations in other settings. Further investigation into renewable energy integration and water treatment innovations within dialysis could also provide additional sustainability benefits.

Overall, this study contributes to a growing evidence base supporting more sustainable practices in kidney care, and highlights the role of procurement and system design in reducing the environmental burden of haemodialysis.

While OpenLCA is a robust and widely used platform for life cycle assessment, it has several limitations. The software relies on background databases (e.g., Ecoinvent) that may contain regionally generalised or outdated data, which can limit the precision of localised environmental modelling. Additionally, OpenLCA uses predefined impact assessment methods and fixed process assumptions, which can constrain flexibility when modelling complex or unique medical systems. These factors may introduce uncertainties, particularly in processes such as waste treatment or transport logistics where real-world variations are not fully captured. Despite these limitations, OpenLCA remains appropriate for comparative analyses like this study, where the relative difference between two systems is the primary focus.

## Conclusion

The Fresenius Granumix system offers a promising alternative to traditional acid concentrate bags in haemodialysis. Significant reductions have been made in environmental impact across multiple categories. As the healthcare sector continues to seek more sustainable practices, systems like Granumix could play a crucial role in reducing the environmental burden of life saving treatments such as haemodialysis.

## Electronic supplementary material

Below is the link to the electronic supplementary material.

Supplementary file1 (DOCX 17 kb)

## Figures and Tables

**Fig. 6:**
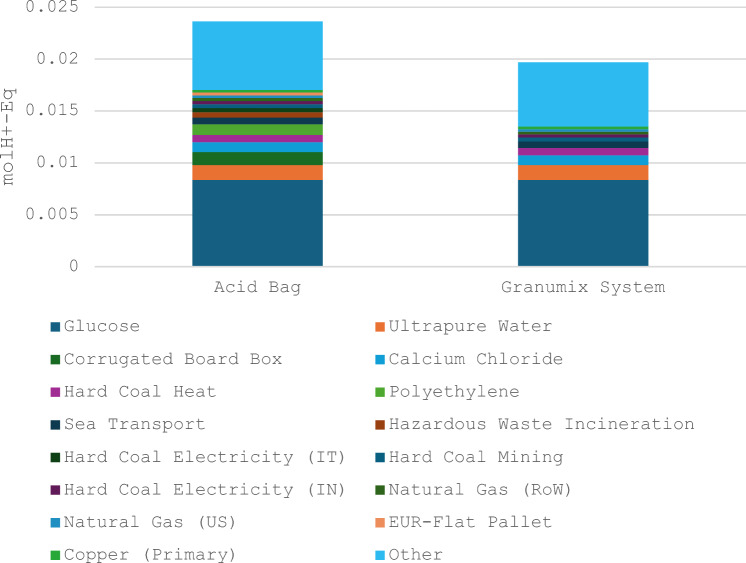
Contribution Analysis, Acidification

**Fig. 7:**
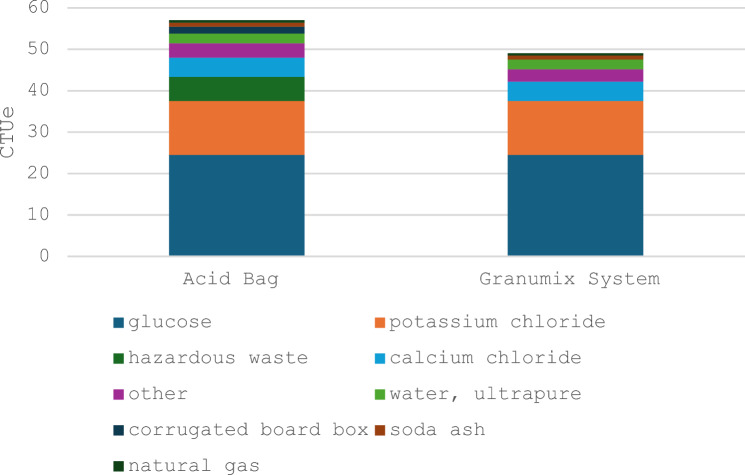
Contribution Analysis, Ecotoxicity

**Fig. 8:**
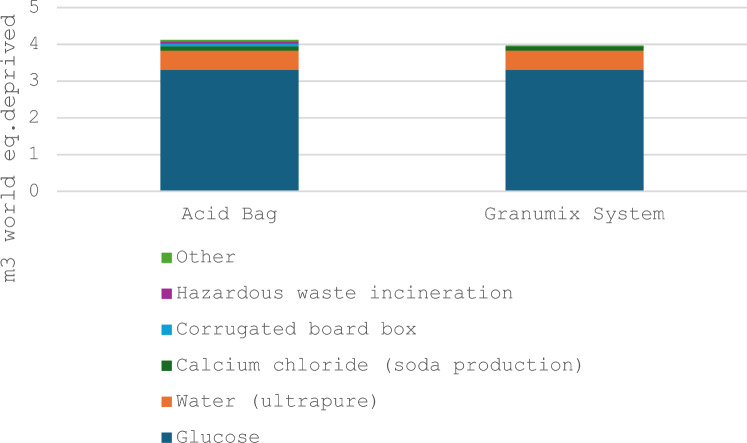
Contribution Analysis, Water Use

**Fig. 9:**
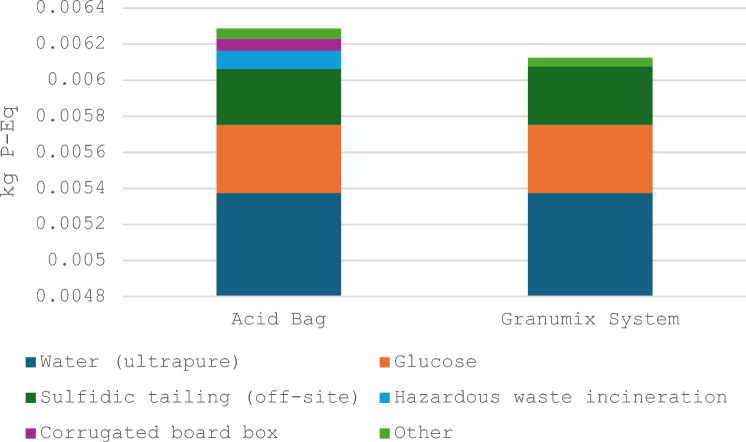
Contribution Analysis, Eutrophication: Freshwater

**Fig. 10:**
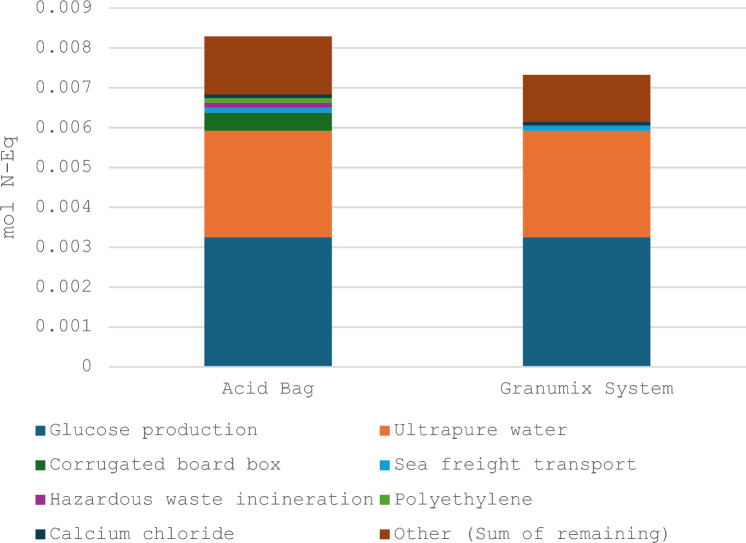
Contribution Analysis, Eutrophication: Marine

**Fig. 11:**
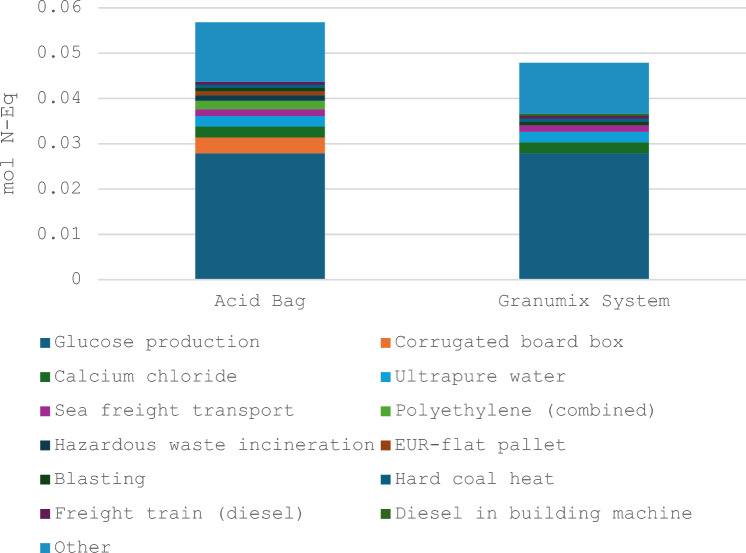
Contribution Analysis, Eutrophication: Terrestrial

**Fig. 12:**
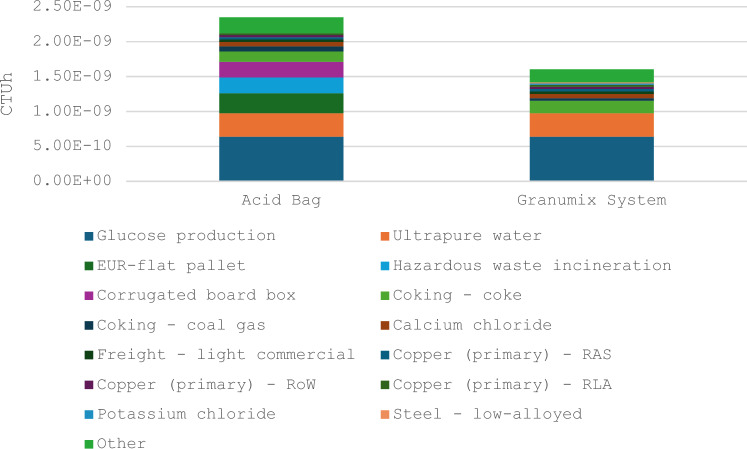
Contribution Analysis, Human Toxicity: Carciogenic

**Fig. 13:**
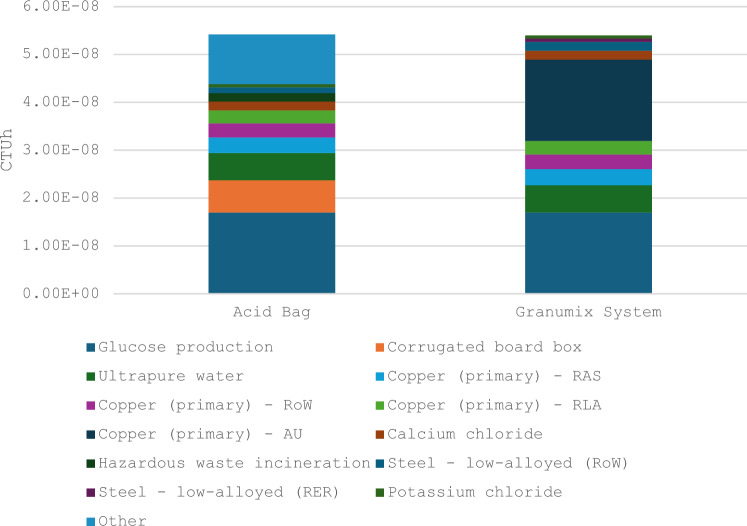
Contribution Analysis, Human Toxicity: Non-Carciogenic

**Fig. 14:**
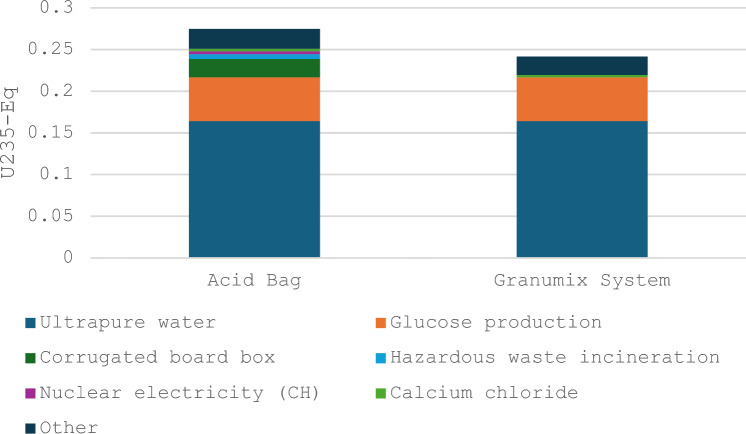
Contribution Analysis, Ionising Radiation

**Fig. 15:**
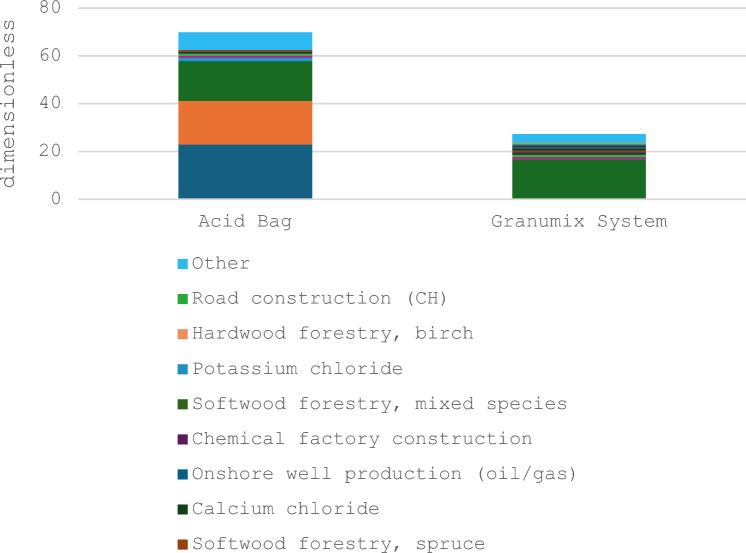
Contribution Analysis, Land Use

**Fig. 16:**
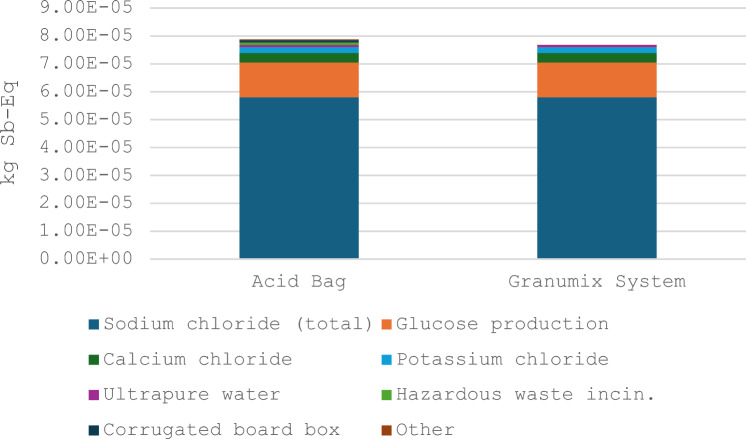
Contribution Analysis, Material Resources: Metals/Minerals

**Fig. 17:**
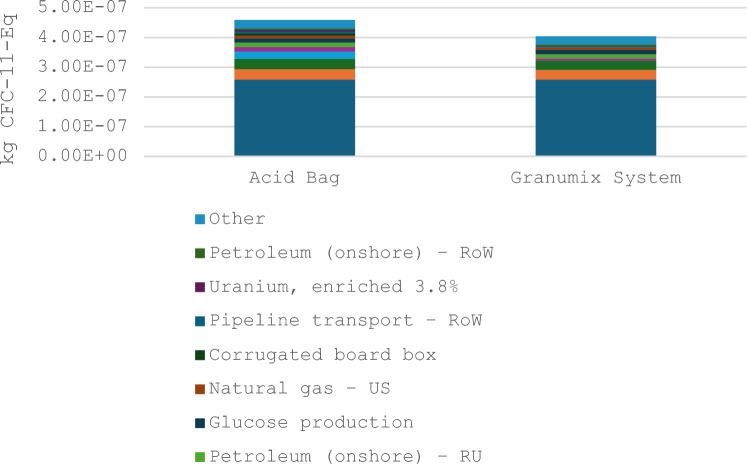
Contribution Analysis, Ozone Depletion

**Fig. 18:**
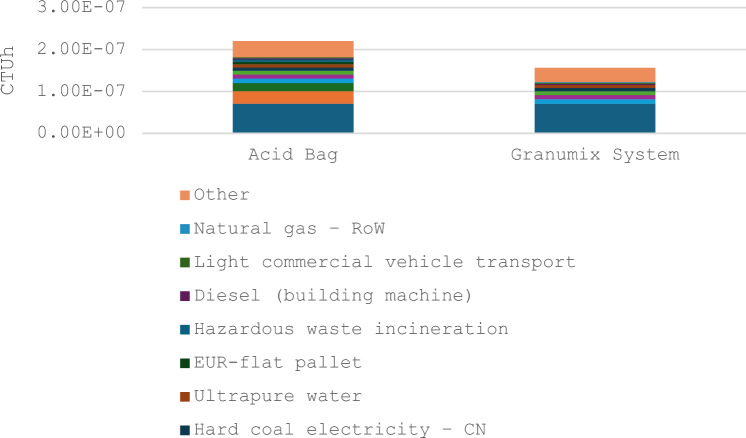
Contribution Analysis, Particulate Matter Formation

**Fig. 19:**
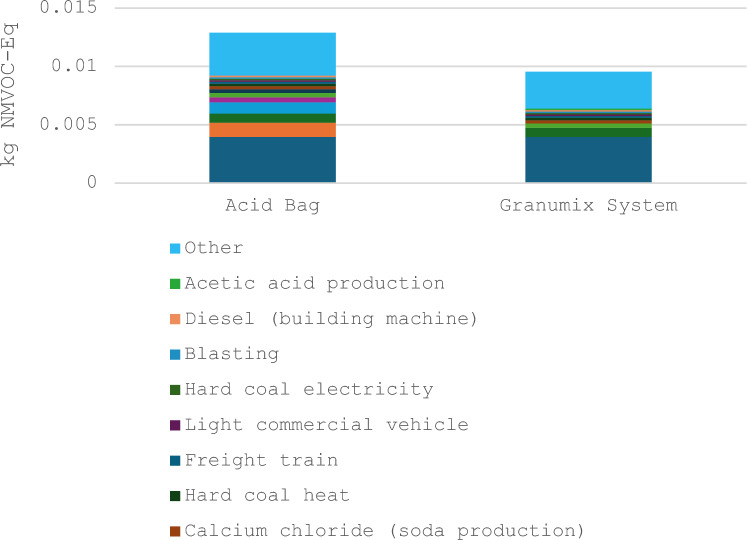
Contribution Analysis, Photochemical Ozonet Formation

## Data Availability

All data generated or analysed during this study are publicly available in the Zenodo repository: Larkin, J. (2025). *Green Haemodialysis: Comparison of Dialysis Bags Versus Fresenius Granumix Water Recycling System at the AOU Policlinico di Modena, Italy* (Version v1). Zenodo. 10.5281/zenodo.14610055.

## Appendix

Appendix 1

See Table 2

**Table 2:** Fresenius Granumix System Building Contribution Table

Category	Inputs / Outputs	Pre-Granumix (acid bags)	Post-Granumix (Granumix System)	Units
Materials	Plastic	Acid concentrate bags: 4500 kg total (0.15 kg × 30,000 bags)	Diamix containers: 867 kg total (0.1445 kg × 6000 containers; each container reused ~ 10 times)	kg/year
	Polypropylene	Data not applicable	Data obtained from Fresenius (used in machine build)	kg/year
	Chemicals	Acid solution: 135,000 L (4.5 L × 30,000 bags)	Diamix concentrate: 1,158,000 L (193 L × 6000 containers)	litres/year
	Cardboard	5367 kg (0.1789 kg × 30,000 units)	1055 kg (0.1758 kg × 6000 units)	kg/year
	Wood (Pallets)	3750 kg total (25 kg × 150 pallets used for 30,000 bags)	720 kg total (25 kg × 28.8 pallets used for 6000 containers)	kg/year
	Materials used in machine build	Not applicable	Stainless steel (208 kg), brass (5 kg), polypropylene (6 kg), polycarbonate (1.5 kg), ABS (4 kg), polyethylene (0.5 kg) – based on manufacturer’s manual and part estimates	kg (one-time build)
	Filter Components	Not applicable	Polysulfone (64.08 g), Polypropylene (15.6 g), Polyurethane adhesive (230.46 g), LDPE packaging (16.71 g), total transport: 199.8 kg*km per filter. Three filters replaced annually	g/filter + transport
Energy	Manufacturing Energy	Estimated at 1.2 MJ per kg of plastic and 1.5 MJ per litre of acid solution: Total ~ 95,000 kWh/year	Estimated at 1.1 MJ per litre of Diamix concentrate: Total ~ 100,000 kWh/year	kWh/year
	Operational Energy	Not applicable	Granumix system power supply: Max 6.4 kWh (380–400 V, 16 A). Energy needs based on Granumix brochure	kWh/year
Water	Water Used in Production	Estimated at 7 L of process water per litre of acid solution: ~ 945,000 L/year (7 L × 135,000 L acid)	Estimated at 5 L of process water per litre of Diamix: ~ 5,790,000 L/year (5 L × 1,158,000 L)	litres/year
	Ultrapure Water for Dialysate Mixing	Not applicable	547,500 L/year (500 L × 3 times/day × 365 days)	litres/year
Waste	Plastic Waste	4500 kg from used acid bags	867 kg from used Diamix containers (each reused ~ 10 times)	kg/year
	Cardboard Waste	5367 kg from bag packaging	1055 kg from container packaging	kg/year
	Wood Waste	3750 kg from used pallets	720 kg from used pallets	kg/year
	Residual Acid Solution	12,000 L total, based on an estimated average of 0.4 L of unused acid concentrate per bag, derived from observational data at the AOU Policlinico di Modena [6]	2000 L total (0.33 L residual per 6000 containers)	litres/year
Recycling / Reuse	Container Reuse	Not applicable	Diamix containers reused up to 10 times before recycling	–
Manufacturing Site	Location	Zona Industriale, Baragiano PZ, Italy	Hafenstraße 9, Schweinfurt, Germany	

## Appendix 2

See Table 3

**Table 3:** Fresenius Granumix System Build Contribution Table

Fresenius Granumix System Build Contribution Table			
Contribution [%]	Process	Total result [kg CO2-Eq]	Unit
0.79 (rest of contribution is from use of machine)	Fresenius Granumix Machine Build (Total Contribution)	0.02086227	
0.57	Steel	0.01502094	kg
0.11	Sheet rolling	0.00293903	kg
0.03	Brass	8.16E-04	kg
0.02	abs	5.88E-04	kg
0.02	Injection moulding	5.41E-04	kg
0.02	Polypropylene	4.60E-04	kg
0.02	Polycarbonate	4.47E-04	kg
0.00	Polyethylene	3.77E-05	kg
0.00	Casting of brass	1.19E-05	kg
0.00	Transport, freight, lorry	1.21E-06	t*km
0.00	Plastic waste	1.00E-03	kg
0.00	Metal waste	1.00E-03	kg

## Appendix 3

Contribution Graphs.

See Figs. 4, 5, 6, 7, 8, 9, 10, 11, 12, 13, 14, 15, 16, 17, 18, 19

## Abbreviations

ABS: Acrylonitrile Butadiene Styrene
AOU: Azienda Ospedaliero-Universitaria
CTUh: Comparative Toxic Units for humans
CTUe: Comparative Toxic Units for ecosystems
ESKD: End Stage Kidney Disease
HD: Haemodialysis
KRT: Kidney Replacement Therapy
LCA: Life Cycle Assessment
LDPE: Low Density Polyethylene
MJ: Megajoule
NH_3_: Ammonia
NMVOC: Non-Methane Volatile Organic Compounds
NO_x_: Nitrogen Oxides
PLA: Polylactic Acid
PM2.5: Particulate Matter smaller than 2.5 microns
Sb-eq: Antimony Equivalent
SO_2_: Sulphur Dioxide
U235-eq: Uranium-235 Equivalent
