# Supplementary material for: Green haemodialysis: comparison of dialysis bags versus fresenius granumix at the AOU Policlinico di Modena, Italy
Source: J Nephrol. 2025 Nov 1;38(9):2619–39. doi: 10.1007/s40620-025-02416-0 (PMC12712103; doi:10.1007/s40620-025-02416-0)
Supplement: Supplementary file 1 — Supplementary file1 (DOCX 17 kb) [file 40620_2025_2416_MOESM1_ESM.docx]

# **Supplementary Material S1**

# Glossary of Life Cycle Assessment (LCA) Impact Categories

This glossary provides definitions and units for the environmental impact categories used in the life cycle assessment (LCA) of acid delivery methods in haemodialysis. These categories are based on standard LCA methodology and reflect a broad range of environmental and human health effects [31].

**1. Climate Change (Global Warming Potential)**
**Unit:** kg CO₂-equivalent (kg CO₂-eq)
Refers to the contribution of greenhouse gases to global warming, expressed in terms of carbon dioxide equivalents.

**2. Acidification Potential**
**Unit:** mol H⁺-equivalent (mol H⁺-eq)
Measures the emission of acidifying substances (e.g., sulfur dioxide, nitrogen oxides) that contribute to acid rain and soil/water acidification, affecting ecosystems and buildings.

**3. Eutrophication Potential**
**Units:**

- Freshwater: kg phosphorus-equivalent (kg P-eq)
- Marine: kg nitrogen-equivalent (kg N-eq)
- Terrestrial: mol nitrogen-equivalent (mol N-eq)
  Assesses nutrient enrichment in water or soil systems, which can cause harmful algal blooms, oxygen depletion, and ecosystem disruption.

**4. Human Toxicity Potential**
**Unit:** Comparative Toxic Units for humans (CTUh)
Estimates potential harm to human health from exposure to toxic substances, both carcinogenic and non-carcinogenic, across the life cycle of a product or process.

**5. Freshwater Ecotoxicity**
**Unit:** Comparative Toxic Units for ecosystems (CTUe)
Represents the potential for chemical emissions to affect freshwater aquatic organisms.

**6. Ionising Radiation**
**Unit:** kilobecquerel uranium-235-equivalent (kBq U235-eq)
Evaluates the impact of radioactive substances on human health.

**7. Land Use (Soil Quality Index)**
**Unit:** Dimensionless score
Assesses changes in land occupation and transformation that affect biodiversity, soil quality, and ecosystem services.

**8. Resource Depletion: Mineral and Metals**
**Unit:** kg antimony-equivalent (kg Sb-eq)
Quantifies the depletion of non-renewable mineral resources.

**9. Ozone Depletion Potential**
**Unit:** kg CFC-11-equivalent (kg CFC-11-eq)
Estimates the potential of emissions to deplete the stratospheric ozone layer.

**10. Particulate Matter Formation**
**Unit:** Disease incidence
Assesses the potential impact of fine particles (PM2.5) on human respiratory health.

**11. Photochemical Ozone Formation**
**Unit:** kg Non-Methane Volatile Organic Compounds-equivalent (kg NMVOC-eq)
Estimates the formation of ground-level ozone (smog) from reactions between VOCs and nitrogen oxides under sunlight.

**12. Water Use**
**Unit:** Cubic metres world equivalent deprived (m³ world eq. deprived)
Reflects the potential deprivation of freshwater resources due to water consumption, relative to local water scarcity.

# **Supplementary Material S2**

Larkin, J. (2025). Green Haemodialysis: Comparison of Dialysis Bags Versus Fresenius Granumix Water Recycling System at the AOU Policlinico di Modena, Italy (Version v1). Zenodo. <https://doi.org/10.5281/zenodo.14610055>
